# Insights into genomic sequence diversity of the SAG surface antigen superfamily in geographically diverse *Eimeria tenella* isolates

**DOI:** 10.1038/s41598-024-77580-7

**Published:** 2024-11-01

**Authors:** Alice Li-Wen Kiang, Shu-San Loo, Mohd-Noor Mat-Isa, Chyan-Leong Ng, Damer P. Blake, Kiew-Lian Wan

**Affiliations:** 1 Department of Biological Sciences and Biotechnology, Faculty of Science and Technology, Universiti Kebangsaan Malaysia, 43600 Bangi, Selangor DE Malaysia; 2 School of Biosciences, Faculty of Science and Engineering, University of Nottingham Malaysia, 43500 Semenyih, Selangor DE Malaysia; 3 Malaysia Genome and Vaccine Institute, National Institutes of Biotechnology Malaysia, Jalan Bangi, 43000 Kajang, Selangor DE Malaysia; 4 Institute of Systems Biology, Universiti Kebangsaan Malaysia, 43600 Bangi, Selangor DE Malaysia; 5 Pathobiology and Population Sciences, Royal Veterinary College, North Mymms, Hertfordshire, AL9 7TA UK

**Keywords:** Coccidiosis, Single nucleotide polymorphism, Natural selection, Avian disease, Genome informatics, Parasitic infection, Comparative genomics, Parasite genomics

## Abstract

*Eimeria tenella* is among the protozoan parasites that cause the infectious disease coccidiosis in chickens, incurring huge economic losses to the global poultry industry. Surface antigens (EtSAGs) involved in host-parasite interaction are potential targets for control strategies. However, the occurrence of genetic diversity for EtSAGs in field populations is unknown, as is the risk of such diversity to the efficacy of EtSAG-based control approaches. Here, the extent of EtSAG genetic diversity and its implications on protein structure and function is assessed. Eighty-seven full-length EtSAG genomic sequences were identified from *E. tenella* genome assemblies of isolates sampled from continents including North America (United States), Europe (United Kingdom), Asia (Malaysia and Japan) and Africa (Nigeria). Limited diversity was observed in the EtSAG sequences. However, distinctive patterns of polymorphism were identified between EtSAG subfamilies, suggesting functional differences among these antigen families. Polymorphisms were sparsely distributed across isolates, with a small number of variants exclusive to specific geographical regions. These findings enhance our understanding of EtSAGs, particularly in elucidating functional differences among the antigens that could inform the development of more effective and long-lasting anticoccidial control strategies.

**Supplementary Information:**

The online version contains supplementary material available at 10.1038/s41598-024-77580-7.

## Introduction

With the global human population projected to reach 9.7 billion in 2050, addressing issues related to food security and sustainability is of paramount importance^1^. Poultry serves as an important source of animal-derived protein^2^, but its production has consistently grappled with challenges posed by infectious diseases such as coccidiosis, caused by protozoan parasites of the genus *Eimeria*. Coccidiosis compromises food conversion rates in poultry, leading to problems such as nutrient malabsorption, weight loss, and in severe cases, haemorrhage or death^3^. *Eimeria* infection can also predispose to bacterial diseases such as necrotic enteritis, caused by *Clostridium perfringens*^4^. The economic burden of coccidiosis on a global scale has been estimated to exceed £10.4 billion every year, necessitating urgent measures to control its occurrence^5^.

Control of coccidiosis in chickens relies primarily on routine chemoprophylaxis using ionophores or chemical anticoccidial drugs, but the emergence of drug resistance can prove limiting^6–9^. Furthermore, growing public and legislative pressure discourages the use of anticoccidial drugs, with some being banned in the European Union^10^. Alternatives include vaccination using complex formulations of multiple live virulent or attenuated *Eimeria* species, although uptake in many sectors has been limited due to restricted manufacturing capacity and relatively high production costs^11,12^. Recognition of the limitations inherent in current anticoccidial controls continues to fuel demand for more cost-effective solutions. Several *Eimeria* proteins have been identified as candidates for development in novel control strategies, the majority of which are involved in host-parasite interaction and innately exposed to the host immune response^13^.

Ten *Eimeria* species are now known to infect chickens, with *Eimeria tenella* the most extensively studied due to its high prevalence and virulence^14–16^. The principal surface antigen family of *E. tenella* are glycosylphosphatidylinositol (GPI)-linked surface proteins known as EtSAGs^17^, expressed on the surface of invasive zoite life cycle stages^18^. Several EtSAG members have demonstrated the ability to bind to mammalian cells and modulate the host immune response, implying possible roles in host attachment and immune evasion^19,20^. While their exact functions remain unclear, EtSAGs have shown promise as control candidates^21^. However, the use of immunoprotective surface antigens expressed by apicomplexan parasites as vaccine candidates has frequently been undermined by extensive pre-existing genetic diversity in field populations, representing naturally occurring genetic resources for immune escape. This has been observed in *Plasmodium falciparum* where allelic variations in the sequences coding for merozoite surface antigen 1 (MSP1) and apical membrane protein 1 (AMA1) can mediate escape from vaccine-induced immunity^22,23^. Thus, understanding the pre-existing sequence diversity of antigens proposed as anticoccidial vaccine candidates is an important screening step in development to support longevity of future controls.

Studies of genetic diversity within *E. tenella* populations have been scarce. A small genome-wide panel of single-nucleotide polymorphism (SNP) markers were developed and used to assess population structure, suggesting the occurrence of regional variation^24^. Phenotypic studies of the efficacy of immune protection induced by *E. tenella* against challenge by different *E. tenella* strains have revealed examples of antigenic diversity and incomplete cross-protection^25–27^. Studies on specific antigens, such as Apical Membrane Antigen 1 (AMA1), have revealed the occurrence of limited genetic diversity, with distinct, region-specific signatures of selection^24^. Understanding the extent of naturally occurring genetic diversity within EtSAG sequences can inform their future development as vaccine candidates. Previous studies with EtSAGs have used fragmented gene sequences^28,29^. Here, we describe the use of *E. tenella* genome sequence assemblies from isolates obtained in the United States, the United Kingdom, Malaysia, Japan and Nigeria to identify full-length EtSAG loci and assess genetic diversity across the EtSAG repertoire.

## Methods

### Eimeria tenella genome sequence assembly

The genome sequence assembly of the reference *E. tenella* Houghton (EtH) strain from the United Kingdom was obtained from ToxoDB^18^. Raw reads of the Wisconsin strain (EtWis) from the United States^30^, the Nippon-2 isolate (EtNt-2) from Japan and the Nigeria-05 isolate (EtNg05) from Nigeria were accessed from the NCBI sequence read archive (SRA) (BioProject PRJEB4009), under the accession numbers ERX270210, ERX269874 and SRX23839148, respectively. The Negeri Sembilan-6 (EtNSN6) and Selangor-6 (EtSGR6) isolates were sampled in Malaysia, and their genomes sequenced as previously reported^31,32^. The Malaysian sequencing reads are available from the NCBI SRA (BioProject PRJNA1085519), under the accession numbers SRX23878533 and SRX23880145, respectively. Raw sequencing reads of EtNSN6, EtSGR6, EtNt-2, EtWis, and EtNg05 were trimmed using Sickle v1.33^33^, followed by PhiX contaminant removal using BBDuk v35.85 (sourceforge.net/projects/bbmap). Cleaned reads of each isolate were aligned to the EtH reference genome using BWA-MEM v0.7.12^34^. Alignment output SAM files were converted into BAM files using the view command in SAMtools suite v1.9^35^, then aligned sequences were sorted with leftmost coordinates using the sort command, and paired-end and single reads were merged into a single file using the merge command. Consensus sequences were then generated using BCFtools v1.9^36^, with assembly quality accessed using the apicomplexa_odb10 reference set of BUSCO 5.4.4^37^.

### EtSAG identification and comparative analysis

Putative EtSAG coding sequences were identified from genome assemblies representing each isolate using EtH SAG protein sequences obtained from ToxoDB^18^ as reference via the Exonerate v2.2.0 protein2genome module^38^. The alignment output files were transformed into GFF3 format using a Python script. From each genome, BEDtools v2.27.1 was used to extract the exons, introns, and inter-coding regions, which are DNA sequences located between the coding regions of two successive genes^39^. Subsequently, pairwise comparisons were performed between the exon, intron and inter-coding region sequences of each Et isolate and EtH EtSAGs using the Exonerate v2.2.0 affine: local module^38^.

### Variant calling

Filtered reads of each isolate, including paired reads and singletons, were mapped against the reference EtH genome using BWA-MEM v0.7.12^34^. Alignment files generated from the mapping were fixed, sorted and merged using SAMtools v1.9 utilities^35^, and duplicates were marked using Picard v2.21.4 (http://broadinstitute.github.io/picard). Variant calling and filtering were performed using BCFtools v1.9 by removing single nucleotide polymorphisms (SNPs), and insertions and deletions (InDels) of quality scores lower than 20. Depth of coverage for each variant was also filtered to fall within the respective minimum and maximum threshold for each *E. tenella* isolate^36^. Classification and annotation of the variants were performed using SnpEff v4.1 g^39^. Variants within the exons, introns and inter-coding regions of EtSAG genes were retrieved based on gene coordinates addressed in the GFF3 file using BEDtools v2.27.1 intersectBed toolset^40^.

### Genetic analysis

Additional EtSAG sequences representing *E. tenella* isolates from China, Korea and India were obtained from the GenBank database to ensure a more comprehensive genetic analysis. The sequences included Korea EtSAG1 (*n* = 101; MZ576739 to MZ576839), China EtSAG1 (*n* = 20; KY117193.1 to KY117212.1), China EtSAG5 (*n* = 1; EF635426.1), China EtSAG10 (*n* = 2; EF649989.1 and EU378908.1), China EtSAG14 (*n* = 1; EF649988.1) and India EtSAG1 (*n* = 2; KF718807.1 and KF718808.1). The number of segregating sites (S), haplotypes (H), haplotype diversity (Hd), and nucleotide diversity (π) were claculated using DnaSP v6^41^. The rates of non-synonymous (dN) and synonymous (dS) substitution were computed using the Z-test (*p* < 0.05) in MEGA6^42^, employing a model based on Nei and Gojobori’s method^43^ with Jukes and Cantor (JC) correction and applying 1000 bootstrap replication. To evaluate the neutral theory of natural selection in the exons and introns, Tajima’s D^44^, and Fu and Li’s D* and F*^45^ statistical tests were performed using DnaSP v6. A sliding window of 100 bp was applied to identify putative regions with evidence of selective sweeps.

### Protein structure prediction

Two EtSAGs, EtSAG1 and EtSAG10, were most highly represented within our sample set and selected for 3D structure prediction using AlphaFold2^46^ in the ColabFold web interface^47^. Distinct amino acid haplotypes were identified using DnaSP for each EtSAG with one representative of each chosen for 3D protein structure prediction. To enhance accuracy of the structure prediction, a template search against PDB100 database was conducted. Subsequently, structural superposition was performed among isolates of EtSAG1 and EtSAG10 using ChimeraX v1.3^48^ with an iteration cutoff of 2.0 Å. Similarities and differences between the reference and polymorphic haplotypes were assessed based on their electrostatic potential and hydrophobicity surfaces.

## Results

### Identification and characterization of EtSAGs

Assembled genomes of EtNSN6, EtSGR6, EtNt-2, EtWis and EtNg05 ranged from 51.85 to 51.88 Mb in size, with a contig N50 size of about 201 kb (Supplementary Table S1). The BUSCO assessment completeness varied between 81.4% and 81.8% based on the apicomplexa_odb10 reference set (Supplementary Table S2). Using Exonerate, a total of 87 EtSAG coding sequences were identified in the reference H genome and from each of the *E. tenella* isolates (Supplementary Table S3). All putative genes identified from the coding regions were predicted to be full-length, ranging from 876 to 1,869 bp. Exons, introns and inter-coding region sequences were analyzed by pairwise comparison with the equivalent EtH EtSAG. The number of exons and introns identified per sequence was consistent for each EtSAG with the reference strain - no exon gain or loss was predicted. Three EtSAGs contained three exons and two introns, 50 EtSAGs contained four exons and three introns, 31 EtSAGs contained five exons and four introns, and three EtSAGs contained six exons and five introns (Supplementary Table S4). The lengths of exons, introns and inter-coding regions were comparable between isolates with averages of 175.7 bp, 153.2 bp and 3319.2 bp, respectively (Table 1). Furthermore, high nucleotide identity was observed in the exons (> 96.3%), introns (> 93.4%) and inter-coding regions (> 99.4%).

**Table 1:** Characteristics of exons, introns and inter-coding regions in EtSAG loci within *E. tenella* isolate genomes. The identity represents the percentage of absolutely-matching nucleotides between reference EtH and respective isolates through pairwise alignment.

Isolate	Exon				Intron				Inter-coding-region			
	Number	Avg Length ± SD (bp)	Identity (%)		Number	Avg Length ± SD (bp)	Identity (%)		Number	Avg Length ± SD (bp)	Identity (%)	
			Min	Max			Min	Max			Min	Max
EtH	382	175.70 ± 101.97	-	-	295	153.20 ± 67.18	-	-	75	3319.21 ± 4373.90	-	-
EtNSN6		175.70 ± 101.97	96.3	100		153.16 ± 67.15	96.9	100		3319.22 ± 4373.85	99.6	100
EtSGR6		175.70 ± 101.97	98.3	100		153.16 ± 67.15	98.8	100		3319.25 ± 4373.90	99.6	100
EtNt-2		175.70 ± 101.97	97.7	100		153.22 ± 67.19	99.0	100		3319.07 ± 4373.70	99.9	100
EtWis		175.70 ± 101.97	97.2	100		153.12 ± 67.09	93.4	100		3319.27 ± 4373.84	99.4	100
EtNg05		175.70 ± 101.97	98.0	100		153.18 ± 67.19	98.7	100		3319.17 ± 4373.90	99.4	100

### Polymorphism patterns between EtSAGs

**Figure 1:**
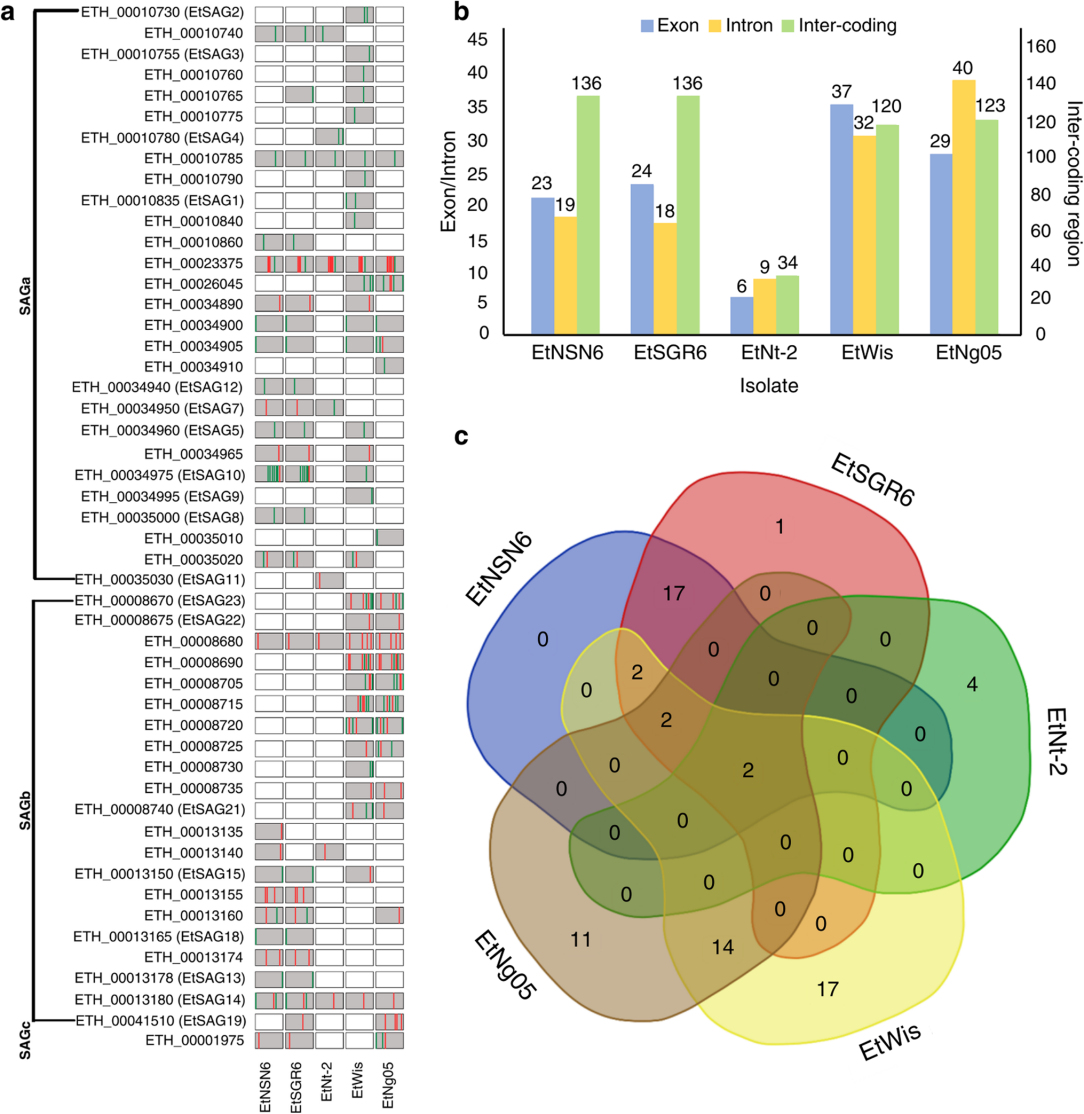
Mutations identified in EtSAG loci within *E. tenella* isolate genomes. (**a**) Mutations in EtSAGs of each isolate. Grey box denotes the presence of mutation in EtSAGs. Each grey box was annotated with mutation in the exons (green), and mutation in the introns (red). (**b**) Overall count of SNPs detected in exons (blue), introns (yellow), and inter-coding regions (green). (c) Distribution of unique and shared SNPs within the exons of *E. tenella* isolates.

Analysis of the EtH genome sequence assembly identified 87 EtSAGs, which were categorized into three sub-families, SAGa (60 members), SAGb (26 members) and SAGc (1 member)^18^. All 87 EtSAGs present in the EtH genome were identified across all *E. tenella* isolate genomes. Comparison with the EtH reference sequence showed that 50 of the predicted 87 EtSAGs displayed polymorphism in their exons and/or introns, distributed across SAGa (28 members, 46.7%), SAGb (21 members, 80.7%) and SAGc (one member, 100.0%) (Fig. 1a). Mutations in the SAGb members (*n* = 10, 47.6%) were mainly detected in both exon and intron sequences, while mutations in the SAGa members (*n* = 19, 67.9%) were predominantly detected in only exon sequences (Supplementary Table S5). Comparison of polymorphic exons and introns between the sub-families revealed SAGc contains the highest polymorphism, averaging one variant per 134 bp, followed by SAGa with one variant per 146 bp, and SAGb with one variant per 160 bp. Specifically, EtSAG10 from the SAGa sub-family exhibited the highest polymorphism, averaging one mutation per 129 bp, mostly in exon sequences (Fig. 1a). On the other hand, amongst the polymorphic EtSAGs, EtSAG13 of SAGb exhibited the lowest polymorphism, with one mutation per 1611 bp. However, a different pattern emerged when considering only the polymorphic exons, with SAGb exhibiting the highest polymorphism (one variant per 149 bp), followed by SAGa (one variant per 160 bp) and SAGc (one variant per 167 bp). Analysis of the genomic sequences showed that introns (one mutation per 151 bp) and exons (one mutation per 156 bp) exhibited similar levels of polymorphism, with inter-coding regions showing comparatively lower variation (one mutation per 747 bp) (Supplementary Table S6). Among EtSAGs that were detected with mutations, nearly two-fifths (*n* = 26) hosted more than one SNP. Notably, SNPs detected in exons were primarily non-synonymous (ns), with approximately 66% (*n* = 46) of them resulting in amino acid changes.

### Polymorphism patterns between isolates

The distribution of mutations across exons, introns and inter-coding regions varied among the five *E. tenella* isolates (Fig. 1b). Comparison of polymorphisms between the isolates revealed that exon SNPs were generally of low frequency and sparsely distributed across isolates from different geographical regions, with approximately half of the SNPs exclusively detected in a single isolate (Fig. 1a). Of the 70 exon SNPs detected in EtSAGs, 17 were unique to EtWis, 11 to EtNg05, four to EtNt-2 and one to EtSGR6 (Fig. 1c). Only two SNPs, T621G and A228G found in two SAGs (ETH_00010785 and ETH_00023375), were shared across all non-reference isolates (Fig. 1a). Additionally, about half of the SNPs (*n* = 18) detected in EtWis and EtNg05 were shared, while over 96% (*n* = 23) of common SNPs were observed between the Malaysian isolates (Fig. 1c), including the non-synonymous mutations detected in SAG10 (Fig. 1a). Similar trends were also noted in the introns and inter-coding regions, with 75% (*n* = 24) of the mutations in the introns and 56% (*n* = 67) in the inter-coding regions detected in EtWis matching those of EtNg05 (Supplementary Fig. S1). Approximately 89% (*n* = 17) of the mutations in introns and 97% (*n* = 132) in inter-coding regions were shared among the Malaysian isolates (Supplementary Fig. S1).

### Genetic analysis of EtSAGs

**Figure 2:**
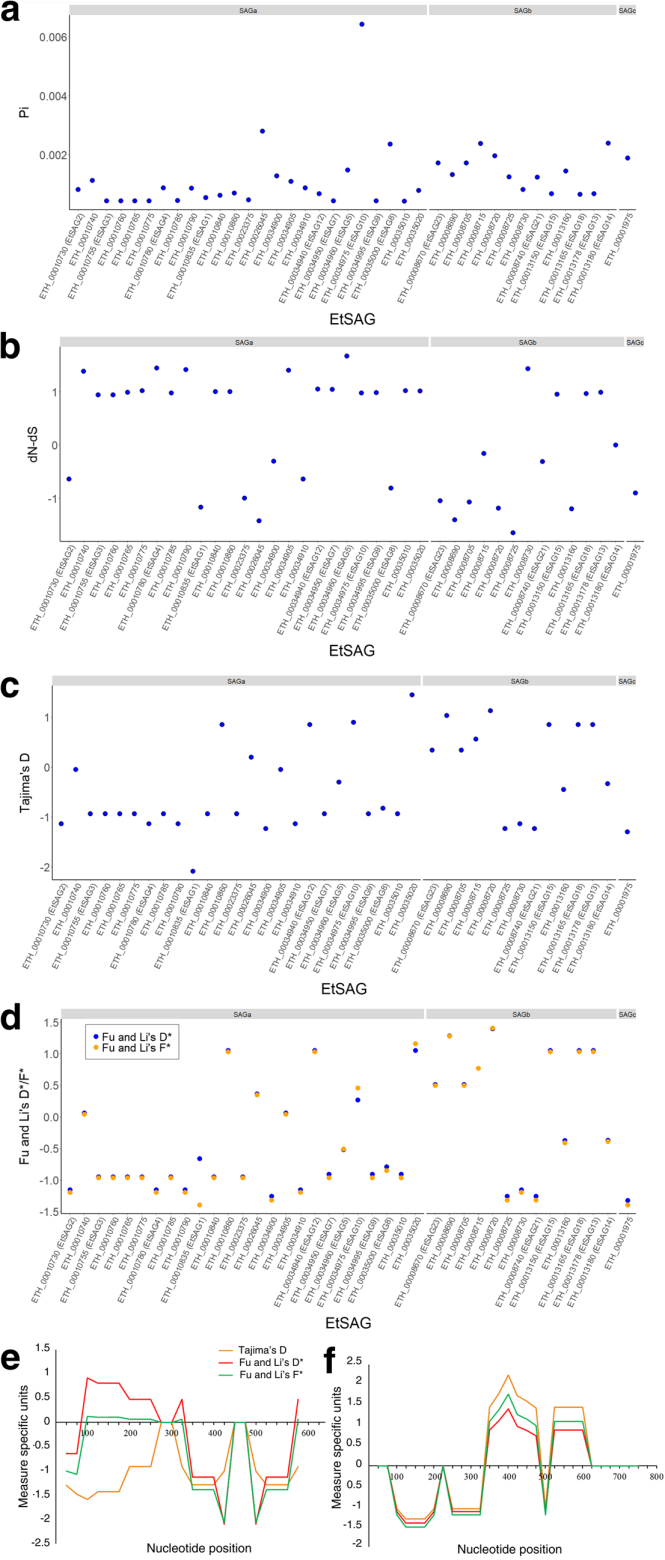
Genetic analyses of EtSAGs. (**a**) Nucleotide diversity; (**b**) Z test; (**c**) Tajima’s D; and (**d**) Fu and Li’s D* (blue dots) and F* (orange dots) analyses in 39 *E. tenella* EtSAGs that displayed mutations in their exons. Sliding window plot analyses of (**e**) EtSAG1 with the total number of sequences included, *n* = 129; (f) EtSAG10 with *n* = 11. Tajima’s D (orange line), Fu and Li’s D* (red line) and Fu and Li’s F* (green line). Window size of 100 bp and 25 bp step size was used.

Interrogation of public sequence repositories uncovered 127 EtSAG sequences from other countries, consisting of Korea EtSAG1 (*n* = 101), China EtSAG1 (*n* = 20), China EtSAG5 (*n* = 1), China EtSAG10 (*n* = 2), China EtSAG14 (*n* = 1) and India EtSAG1 (*n* = 2). Nucleotide diversity was assessed in EtSAGs that exhibited mutations in their exons and introns. Consequently, the analysis was focused on 39 EtSAGs (SAGa, *n* = 25; SAGb, *n* = 13; SAGc, *n* = 1) with mutations in exons and 28 EtSAGs (SAGa, *n* = 9; SAGb, *n* = 18; SAGc, *n* = 1) with mutations in introns. Limited diversity was observed, with SAGa exons having an average nucleotide diversity of 0.00109 ± 0.00116, SAGb 0.00141 ± 0.00059 and SAGc 0.00189 (Fig. 2a). Similar findings were seen in introns, with SAGa, SAGb and SAGc average nucleotide diversity at 0.00174 ± 0.00091, 0.00141 ± 0.00081 and 0.00228, respectively (Supplementary Fig. S2).

The estimated variance of dN-dS values for 39 EtSAGs ranged between − 1.6458 and 1.6772 (SAGa, -1.4188 to 1.6772; SAGb, -1.6458 to 1.44; SAGc, -0.8964), including EtSAG1 and EtSAG10 with an estimated variance of dN-dS values at -1.1678 and 0.9822, respectively (Fig. 2b). SAG14 is the only EtSAG having an estimated variance of dN-dS value close to zero (Fig. 2b). Tajima’s D values ranged between − 2.083 and 1.445 for exons (SAGa, -2.083 to 1.445; SAGb, -1.233 to 1.124; SAGc, -1.295) and between − 1.132 and 1.445 for introns (SAGa, -1.132 to 1.445; SAGb, -1.132 to 1.124; SAGc, -0.05) (Fig. 2c, Supplementary Fig. S2), with no statistical significance. Fu and Li’s D* and Fu and Li’s F* values yielded consistent results. Non-significant Fu and Li’s D* values in exons ranged between − 1.325 and 1.396 (SAGa, -1.260 to 1.052; SAGb, -1.26 to 1.396; SAGc, -1.325) and in introns, they ranged between − 1.155 and 1.396 (SAGa, -1.155 to 1.052; SAGb, -1.155 to 1.396; SAGc, 0.062) (Fig. 2d, Supplementary Fig. S2); while non-significant Fu and Li’s F* ranged between − 1.397 and 1.406 (SAGa, -1.397 to 1.158; SAGb, -1.318 to 1.406; SAGc, -1.396) in exons, and between − 1.195 and 1.406 (SAGa, -1.195 to 1.158; SAGb, -1.195 to 1.406; SAGc, 0.04) in introns (Fig. 2d, Supplementary Fig. S2).

For EtSAG1, with the largest number of representative sequences, no significant evidence of selection was observed using sliding window analysis (Fig. 2e). However, a sliding window analysis of EtSAG10 identified significant Tajima’s D (*p* < 0.05) and Fu and Li’s F* (*p* < 0.02) values of 2.245 and 1.769, respectively, in nucleotide positions 351 to 450 (Fig. 2f).

### Effects of polymorphisms on EtSAG protein structures

**Figure 3:**
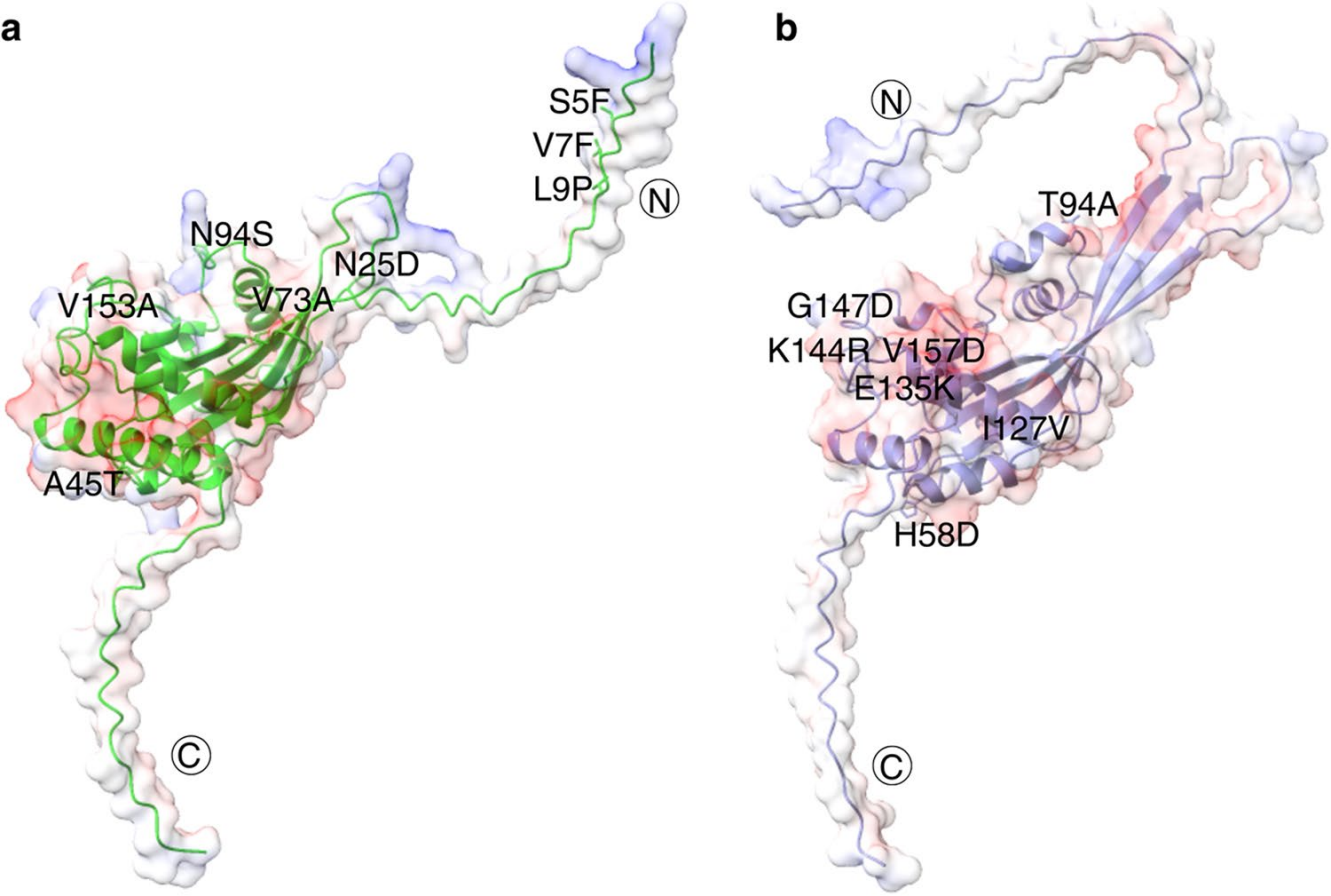
Predicted protein structure of EtSAGs. (**a**) EtSAG1; (**b**) EtSAG10. The red and blue clouds represent the negative and positive surface, respectively. Specific amino acid changes detected in the regions are labeled accordingly.

Eight non-synonymous mutations were identified in EtSAG1 across 129 *E. tenella* sequences from eight different countries, while a total of seven non-synonymous mutations were found in EtSAG10 sequences across eight isolates from six countries (Fig. 3). Protein structure predictions of the haplotypes using AlphaFold2 revealed high confidence in both EtSAG1 and EtSAG10 predictions (Supplementary Fig. S3). In EtSAG1, the mutations S5F and V7F detected in EtNt-2, and the mutation L9P detected in India-2 were located in the N-terminal signal peptide. When compared to EtH EtSAG1, it was predicted that the V7F mutation had no significant effect on the surface hydrophobicity of EtNt-2 EtSAG1. However, substitution of serine to phenylalanine at position 5 is likely to increase the hydrophobicity of the region (Supplementary Fig. S4), in addition the aromatic ring of phenylalanine side-chain may provide additional π–π interaction with its interacting partner. Conversely, the L9P mutation was predicted to result in a reduction in the hydrophobicity of India-2 EtSAG1 (Supplementary Fig. S4). The other mutations were mostly situated on one side of EtSAG1, interconnecting to the N-terminal signal peptide. Notably, the N25D mutation was predicted to impact the electrostatic potential and hydrophilicity of EtWis EtSAG1. The regions encompassing N25 in the EtH EtSAG1 were mostly neutral, but the substitution of asparagine to aspartic acid would likely lead to a strong negative charge surface in the region of EtWis EtSAG1 (Supplementary Fig. S4).

The overall predicated structure of EtSAG10 had a local net negative charge (Supplementary Fig. S5). Non-synonymous mutations found in EtSAG10 include H58D, T94A, I127V, E135K, K144R, G147D and V157A (Table 2), with most of these mutations having no effect on the surface hydrophobicity and electrostatic potential. Nevertheless, comparison with the predicted reference EtH structure suggest that the region surrounding H58D was likely to exhibit a strong hydrophilic and negatively charged surface, while E135K potentially displayed a significant electrostatic potential difference from a negative charge to a strong positive charge surface (Supplementary Fig. S6).

**Table 2:** Non-synonymous SNPs in EtSAG1 and EtSAG10 coding sequences from *E. tenella*.

No	EtSAG1		EtSAG10	
	nsSNP	Isolate	nsSNP	Isolate
1	S5F	EtNt-2	H58D	Yangling
2	V7F	EtNt-2	T94A	Yangling
3	L9P	India-2	I127V	EtNSN6, EtSGR6, Beijing and Yangling
4	N25D	EtWis, India-2, All China and Korea isolates	E135K	EtNSN6, EtSGR6, Beijing and Yangling
5	A45T	MZ576796 and MZ576829	K144R	EtNSN6, EtSGR6, Beijing and Yangling
6	V73A	MZ576750 and MZ576758	G147D	EtNSN6, EtSGR6, Beijing and Yangling
7	N94S	MZ576762 and MZ576800	V157D	Beijing
8	V153A	India-1		

## Discussion

The global poultry industry is experiencing significant growth, but it is also grappling with a substantial economic burden caused by the protozoan parasite *Eimeria*. Understanding genetic and genome-wide diversity for a pathogen can be used to inform on an isolate or population’s ability to escape drug- or vaccine-mediated control, supporting development of effective long-term control strategies. This study aimed to assess the occurrence and extent of genetic diversity within coding and surrounding sequences for the EtSAG gene family.

EtSAGs from diverse geographical regions were found to exhibit a high degree of conservation in their exons, introns and inter-coding regions when compared to the reference EtH SAG sequences. Exon sequence conservation was anticipated as functionally essential regions are often subjected to purifying selection^49^, possibly enhanced by increased DNA repair activity accessibility in these regions^50^. However, similar observations in the non-coding introns and inter-coding regions might suggest otherwise, where the strong conservation in EtSAG loci may be due to the genetic relatedness of the isolates. Genetic variants in EtSAGs occurred randomly, with no discernible patterns among the isolates. However, distinctive SNPs were identified by pairwise comparison between isolates from different geographical regions. For instance, the two Malaysian isolates, whilst sampled from different states of the country, shared a high number of identical variants with most SNPs common to both isolates. Similarly, EtNt-2, EtWis, and EtNg05 displayed similar patterns, with the majority of SNPs unique to each respective isolate, suggesting region-specific polymorphisms. This corresponds with previous findings in EtSAG1 isolates from Korea, China and India with country specific changes^29^. Past studies on genes like *E. tenella* glucose-6-phosphate isomerase (G6-PI), internal transcribed spacer-1 (ITS-1), and apical membrane antigen-1 (AMA-1) have also reported similar strain-defined genetic variations^32,51^.

Comparison of the nucleotide diversity detected in EtSAG sequences identified little or no signatures of selection, with Tajima’s D, and Fu and Li’s D* and F* values showing no significance, suggesting a relatively neutral evolutionary pattern across isolates. This aligns with similar findings in *E. tenella* AMA-1 where limited sequence diversity was reported with minimal purifying selection^24^, supporting its potential as a candidate for anticoccidial vaccine development. Comparison between the SAG families found that SAGb exons exhibit higher polymorphism per bp and nucleotide diversity compared to SAGa exons, despite most SAGb genes showing negative estimated variance of dN-dS values. This observation suggests a potential strategy for antigenic variation during the merozoite stage, as SAGb genes are highly expressed in this stage where recurring cycles of invasion, replication and host cell rupture occurs to release new generations of merozoites, ultimately leading to localized destruction of the intestinal epithelium^18,52^. The higher polymorphism in SAGb might contribute to immune evasion while still preserving essential protein functions. Conversely, SAGa members present in all *Eimeria* species are suggested to be involved in host attachment and immune modulation^18,19^. The positive estimated variance dN-dS values observed in SAGa genes indicate diversifying selection, likely driven by the functional variability and ubiquity of these antigens in host-parasite interactions. Furthermore, analysis of EtSAG10 revealed a positive variance of dN-dS value, suggesting potential adaptation to environmental pressure. Sliding window analysis identified significant positive values of Tajima’s D and Fu and Li’s F* within the region corresponding to nucleotide position 351 to 450, indicating that amino acid changes in EtSAG10 are favored for adaptation, while balancing selection maintains genetic diversity in functionally important regions.

Structural predictions for EtSAG1 and EtSAG10 revealed overall high confidence, with notable variations in the N-terminal signal peptide and C-terminal GPI-anchor, exhibiting lower sequence coverage and pLDDT score at the beginning (~ 20 amino acid) and end (~ 20 amino acid) of the amino acid sequences (Supplementary Fig. S3). This observation aligns with the known intrinsic disorder of the N-terminal and C-terminal tail, which facilitate diverse protein interactions while ensuring process fidelity^53^. All the identified non-synonymous SNPs in EtSAG1 (Table 2) were shown as solvent exposed residues (except A45T) in the predicted EtSAG1 haplotype structures (Fig. 3), suggesting the possible roles in protein-receptor interaction. The mutations observed in the N-terminal signal peptide regions may affect the surface hydrophobicity and electrostatic potential. Signal peptides play a crucial role in protein secretion by directing nascent polypeptides into the export pathway, with the hydrophobicity of the signal peptide being critical for efficient translocation^54^. Increased surface hydrophobicity and presumably additional π–π interaction of aromatic ring from phenylalanine of the S5F mutation in EtNt-2 may improve precursor processing efficacy, potentially leading to enhanced EtSAG1 protein secretion. Moreover, the highly negative-charged surface resulting from the N25D mutation, detected in the majority of *E. tenella* isolates (123 out of 127 isolates) may suggest functional significance for EtSAG1. The well-conserved nature of the N25D mutation among the majority of isolates indicates that it likely confers selective advantage and plays a crucial role in parasite survival.

Similar to the observations in EtSAG1, all the identified non-synonymous SNPs (Table 2) in EtSAG10 were also found as solvent exposed residues in the predicted 3D structure. The majority of mutations detected were found near the C-terminus GPI anchor domain (Fig. 3), which is in close proximity to the plasma membrane of *E. tenella*. This contrast in mutation distribution compared to EtSAG1 suggests potential differences in the functions and selective pressure of EtSAG1 and EtSAG10. While both genes are developmentally expressed, their stage-specific expression patterns are distinctive. EtSAG10 is expressed in sporulated oocysts, sporozoites, and second-generation merozoites, whereas EtSAG1 is only expressed in sporulated oocysts and sporozoites^18^. The expression of EtSAG10 in second-generation merozoites indicates its potential importance in the infectious pathogenic stage. Interestingly, the outward-facing surface of EtSAG10 is predominantly negatively charged compared to EtSAG1 and EtSAG19 (Supplementary Fig. S5) that were reported to have expanded positively charged patch, which is important for sulfated proteoglycans interaction for the parasite to initiate host membrane invasion^20,55,56^.

Structural comparison of EtSAG10, EtSAG1 and EtSAG19 revealed conserved core structures (four-stranded anti-parallel β-sheet surrounded by six α-helices)^56^. Nonetheless, a distinct difference was observed at the N-terminus region. Interestingly, an extended beta-sheet which likely resulted from an insertion event was identified in EtSAG10 (Supplementary Fig. S5). The absence of a positively charged patch and structural differences suggests distinction in EtSAG10 potential function compared to other the other EtSAGs assessed. Additionally, among the two mutations affecting the surface hydrophobicity and charge, the E135K mutation is associated with significantly positive Tajima’s D and Fu and Li’s F* values in that nucleotide position, indicating its potential importance for the structural or functional integrity of the protein. On the other hand, the H58D mutation may be driven by positive selection as demonstrated with a positive estimated variance of dN-dS value, suggesting its potential role in adaptive evolution. The complex adaptive landscape of mutations of EtSAG10 likely represents survival mechanisms of the parasite to thrive in the host environment.

The recent availability of whole genome sequences from diverse *E. tenella* isolates presents an unprecedented opportunity to comprehensively explore the genomic sequence diversity within the EtSAG genes. However, while these isolates represent a range of geographical regions, the limited number of genome sequences may not fully capture the genetic variability of the EtSAG family across global *E. tenella* populations. Future research involving a larger number of isolates from different regions is necessary to gain a more comprehensive understanding of the EtSAG family. Such research would provide deeper insights into their genetic composition and support the development of more effective and durable vaccines.

## Conclusion

This study improves understanding of the genetic diversity of the EtSAG surface antigen superfamily. Low levels of nucleotide diversity within EtSAGs, coupled with geographically specific polymorphisms, underscores the importance of considering regional factors when developing coccidiosis control strategies. The differences in polymorphism and selection pressure between the EtSAG subfamilies, along with the distinctive surface charges and predicted structure also shed light on the potential functional divergence and unique roles for different members of the EtSAG family in the pathogenesis of coccidiosis. These findings lay the groundwork for further investigations into the genetic profile of EtSAGs, necessitating broader studies encompassing a wider spectrum of field isolates from diverse geographical regions. Such comprehensive analyses will enhance understanding of the underlying mechanism driving the diversification of EtSAGs, facilitating the optimization of coccidiosis vaccine control strategies based on these antigens. Ultimately, this can contribute to reducing the significant economic burden imposed by *Eimeria* on the poultry industry worldwide.

## Supplementary information

Below is the link to the electronic supplementary material.

Supplementary Material 1

Supplementary Material 2

## Data Availability

All data supporting the findings of this study are included in this article (and its Supplementary Tables and Figures files). Raw sequence data analyzed in this study are available in the Sequence Read Archive (SRA) under the accession numbers ERX270210 (EtWis), ERX269874 (EtNt-2), SRX23839148 (EtNg05), SRX23878533 (EtNSN6) and SRX23880145 (EtSGR6). All other nucleotide sequences analyzed in this study are publicly available in the GenBank database, under the accession numbers MZ576739-MZ576839 (Korea EtSAG1), KY117193.1-KY117212.1 (China EtSAG1), KF718807.1-KF718808.1 (India EtSAG1), EF635426.1 (China EtSAG5), EF649989.1-EU378908.1 (China EtSAG10), and EF649988.1 (China EtSAG14).
