# Supplementary material for: Insights into genomic sequence diversity of the SAG surface antigen superfamily in geographically diverse Eimeria tenella isolates
Source: Sci Rep. 2024 Nov 1;14:26251. doi: 10.1038/s41598-024-77580-7 (PMC11528073; doi:10.1038/s41598-024-77580-7)

Additional Information

Supportive Figure Legends

Supplementary Figure S1. Distribution of unique and shared EtSAG-associated SNPs among *E. tenella* isolates.

Supplementary Figure S2. Genetic analysis of EtSAGs intronic regions.

Supplementary Figure S3. ColabFold PAE score, sequence coverage and pLDDT score of predicted EtSAG1 and EtSAG10 structure.

Supplementary Figure S4. Predicted protein structure of EtSAG1.

Supplementary Figure S5. Electrostatic distribution of EtSAGs and their structural alignment.

Supplementary Figure S6. EtSAG10 surface representation.

Supplementary Figure S1. Distribution of unique and shared EtSAG-associated SNPs among *E. tenella* isolates. (a) Intron region. (b) Inter-coding region.

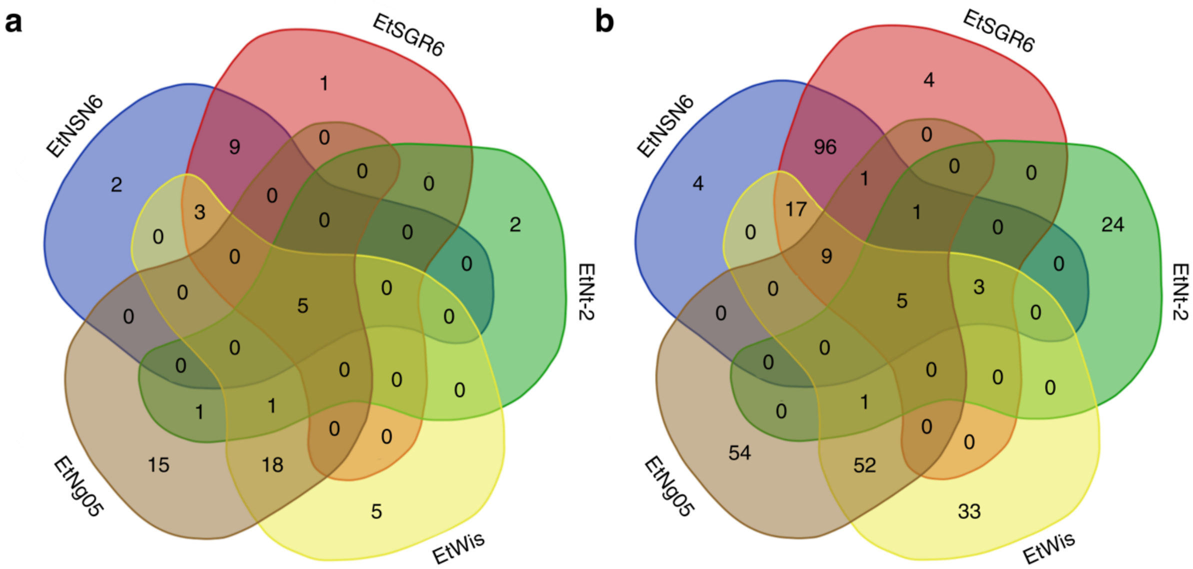

Supplementary Figure S2. Genetic analysis of EtSAGs intronic regions. (a) Nucleotide diversity; (b) Tajima's D; (c) Fu and Li's D* (blue dots) and F* (orange dots) analyses in 28 *E. tenella* EtSAGs that displayed mutations in their introns.

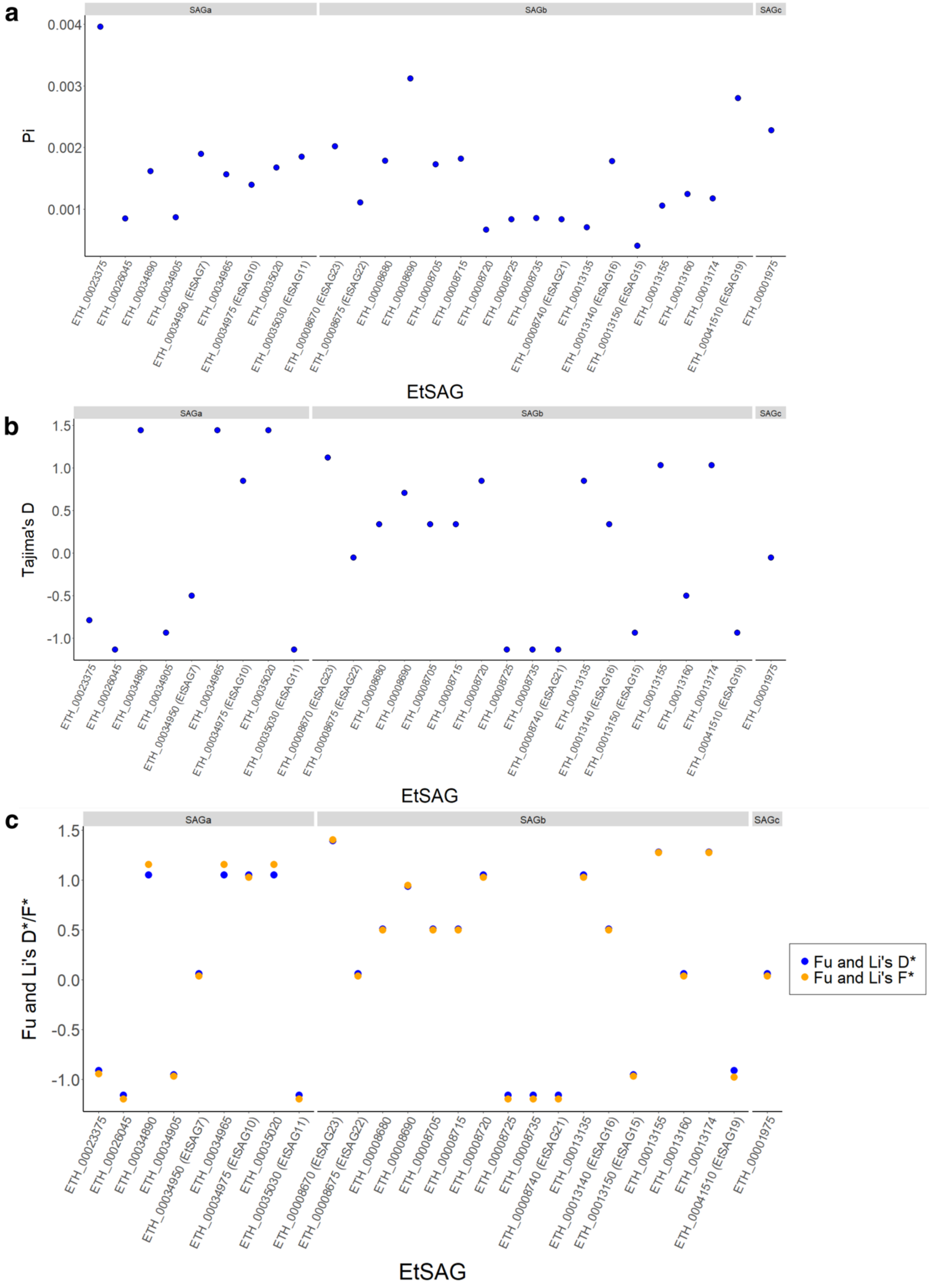

Supplementary Figure S3. ColabFold PAE score, sequence coverage and pLDDT score of predicted reference EtH structure as representative of each EtSAG structure. (a) EtSAG1. (b) EtSAG10.

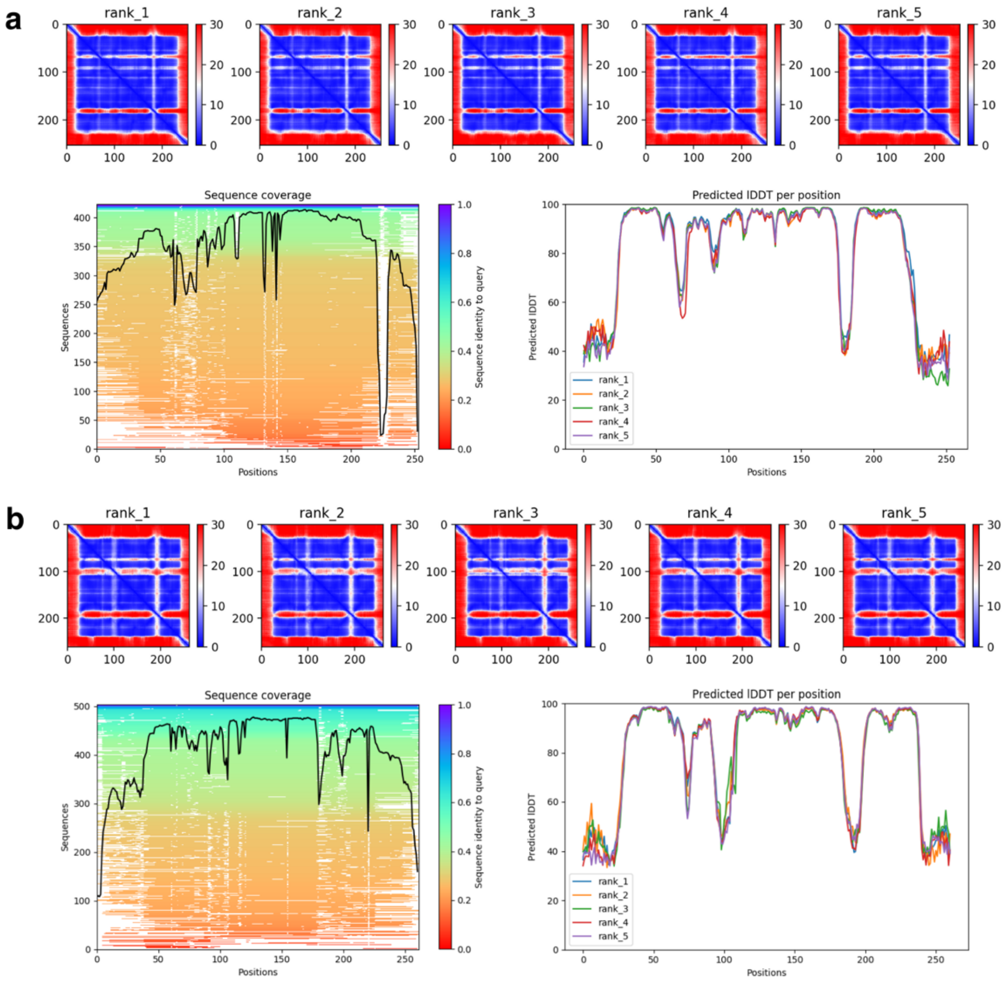

Supplementary Figure S4. Predicted protein structure of EtSAG1. Hydrophobic representation of (a) EtH, (b) EtNt-2 and (c) India-2 EtSAG1 with mutations indicated in the N-terminal signal peptide. Surfaces coloured in dark cyan show the most hydrophilic areas, moving through white to gold showing the most hydrophobic areas. Electrostatic representation of (d) EtH and (e) EtWis EtSAG1, with mutational sites labelled. Surfaces coloured red show negative charges and blue indicates positive charges. The electrostatic ranges set by the program default is -10 to 10.

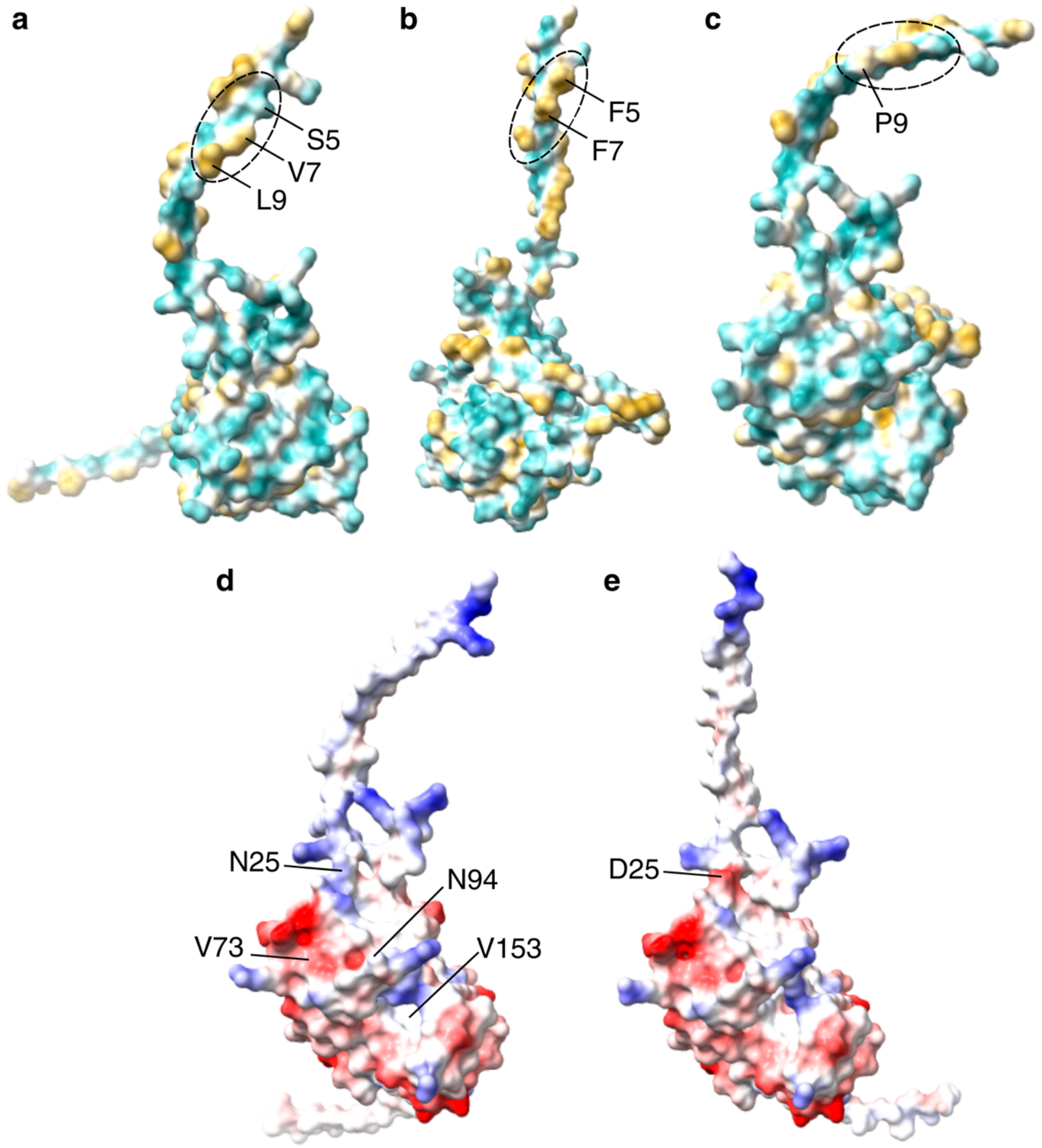

Supplementary Figure S5. Electrostatic distribution of EtSAGs and their structural alignment. (a) Sideview of EtSAG10 predicted electrostatic potential with the surfaces coloured red show negative charges and blue indicates positive charges. The electrostatic ranges set by the program default is -10 to 10. (b) Sideview of EtSAG1 and (c) EtSAG19 with blue patch on one face of the proteins as indicated by dotted circle. (d) Alignment of EtSAG10 (purple), EtSAG1 (green) and EtSAG19 (yellow).

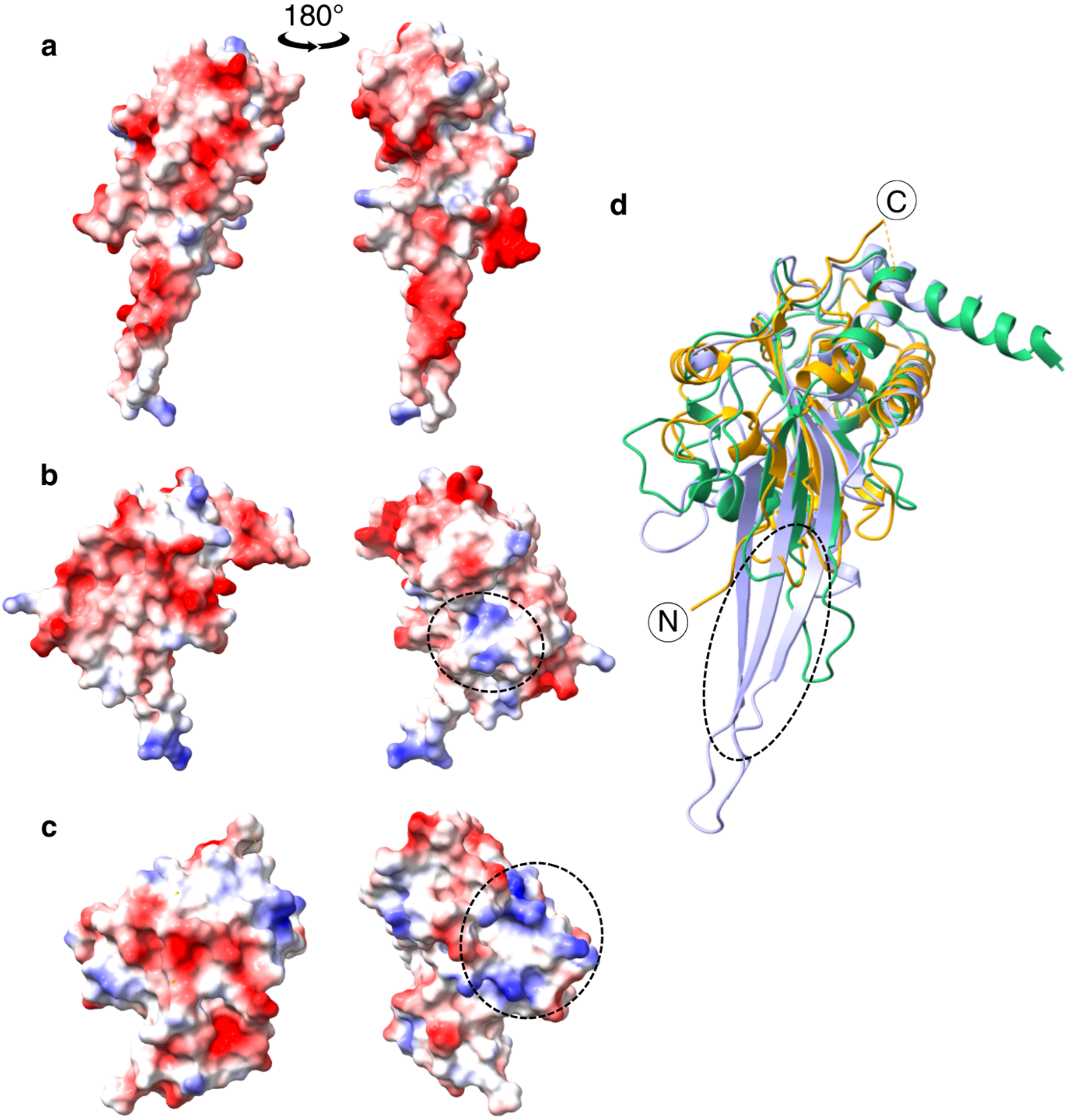

Supplementary Figure S6. EtSAG10 surface representation. (a) Hydrophobic representation of YangLing isolates. Surfaces coloured in dark cyan show the most hydrophilic areas, moving through white to gold showing the most hydrophobic areas. (b) Electrostatic potential of YangLing isolates. Surfaces coloured red show negative charges and blue indicates positive charges. The electrostatic ranges set by the program default is -10 to 10.

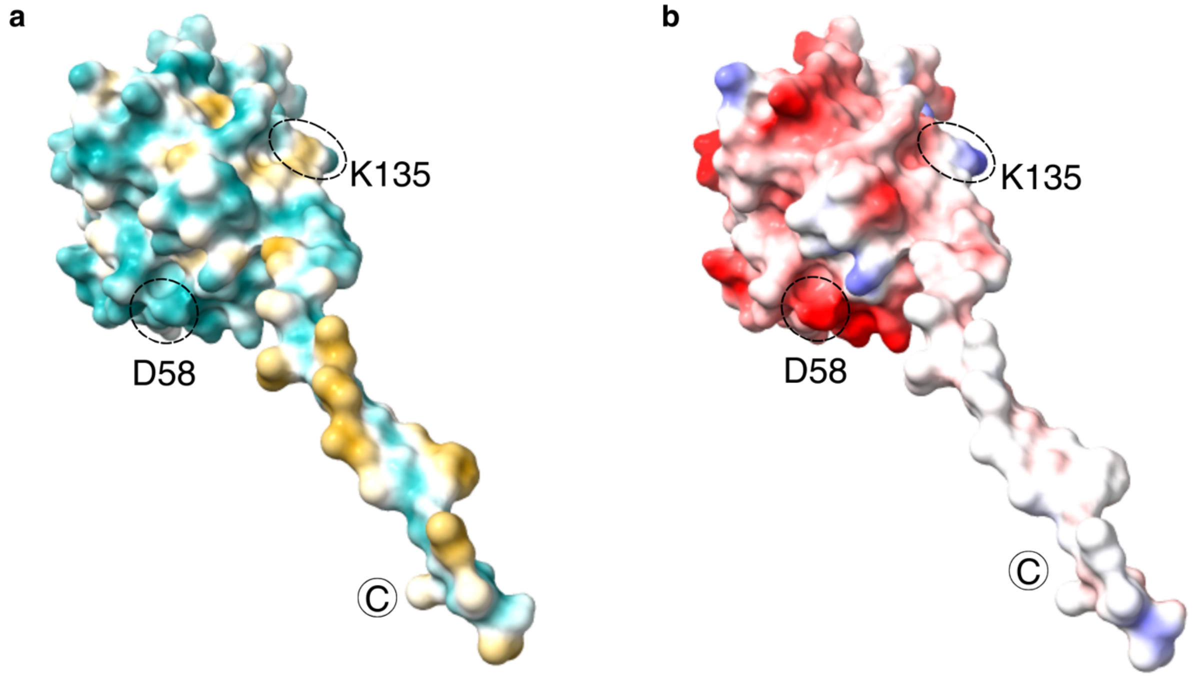

Supplement: Supplementary file 1 — Supplementary Material 1 [file 41598_2024_77580_MOESM1_ESM.pdf]
