# Supplementary material for: Insights into genomic sequence diversity of the SAG surface antigen superfamily in geographically diverse Eimeria tenella isolates
Source: Sci Rep. 2024 Nov 1;14:26251. doi: 10.1038/s41598-024-77580-7 (PMC11528073; doi:10.1038/s41598-024-77580-7)
Supplement: Supplementary file 2 — Supplementary Material 2 [file 41598_2024_77580_MOESM2_ESM.pdf]

Supplementary Table S1. Statistics characterising *E. tenella* isolate genome sequence assemblies

Sample ID	EtNSN6		EtSGR6		EtNt-2		EtWis		EtNg05	
	**Contig**	**Scaffold**	**Contig**	**Scaffold**	**Contig**	**Scaffold**	**Contig**	**Scaffold**	**Contig**	**Scaffold**
Total	12,383	4,664	12,373	4,664	12,700	4,664	12,694	4,664	12,479	4,664
Sum (bp)	51,171,791	51,851,454	51,192,319	51,867,614	51,170,151	51,855,669	51,166,995	51,852,405	51,199,914	51,879,293
N's per 100 kbp	-	1,303	-	1,301	-	1,322	-	1,322	-	1,310
Max size	491,859	1,463,048	491,909	1,463,443	491,836	1,462,681	491,767	1,462,728	491,844	1,463,265
N50	54,962	200,881	54,955	200,946	54,960	200,919	54,960	200,879	54,961	201,030
L50	195	68	195	68	195	68	195	68	195	68
Total > 1 kb	12,383	4,664	12,373	4,664	12,700	4,664	12,694	4,664	12,478	4,663
Sum (bp) > 1 kb	51,171,791	51,851,454	51,192,319	51,867,614	51,170,151	51,855,669	51,166,995	51,852,405	51,198,919	51,878,298
GC content	-	51.32%	-	51.30%	-	51.34%	-	51.33%	-	51.33%

Supplementary Table S2. Assessment of *E. tenella* isolate genome sequence assemblies completeness by BUSCOs using apicomplexa_odb10 reference set

**BUSCOs**	**EtNSN6**		**EtSGR6**		**EtNt-2**		**EtWis**		**EtNg05**	
	**Number**	**Percent (%)**	**Number**	**Percent (%)**	**Number**	**Percent (%)**	**Number**	**Percent (%)**	**Number**	**Percent (%)**
Complete BUSCOs (C)	363	81.4	363	81.4	364	81.6	363	81.4	365	81.8
Complete and single-copy BUSCOs (S)	363	81.4	363	81.4	363	81.4	1362	81.2	365	81.8
Complete and duplicated BUSCOs (D)	0	0	0	0	1	0.2	1	0.2	0	0
Fragmented BUSCOs (F)	58	13	59	13.2	57	12.8	58	13	56	12.6
Missing BUSCOs (M)	25	5.6	24	5.4	25	5.6	25	5.6	25	5.6
Total BUSCOs	446	100	446	100	446	100	446	100	446	100

BUSCO assessment of EtH is 81.1% (derived from Reid et.al 2014).

Supplementary Table S3. Summary of putative SAG coding sequences identified in the genome of each *E. tenella* isolate

Isolate	EtSAGs		
	Total number	Length (bp)	
		Min	Max
EtNSN6	87	876	1868
EtSGR6	87	876	1868
EtNt-2	87	876	1869
EtWis	87	876	1869
EtNg05	87	876	1869

Number of EtSAG genes predicted from each strain sequence assembly, and the minimum and maximum length of the predicted gene.

Supplementary Table S4. Summary of putative *E. tenella* SAG loci exon and intron numbers

Number of exon	Number of intron	Number of EtSAGs	Percentage (%)
3	2	3	3.45
4	3	50	57.47
5	4	31	35.63
6	5	3	3.45

Supplementary Table S5. Summary of EtSAG sub-family characteristics

Sub-family	Total members	Polymorphic members						Number of variants			Polymorphism per bp		
		Overall	length (bp)	Exon	length (bp)	Intron	length (bp)	Exon	Intron	Total	Overall	Exon	Intron
**SAGa**	60	28	8,192	25	6,556	9	1,636	41	15	56	146	160	109
**SAGb**	26	21	11,525	13	4,027	18	7,498	27	45	72	160	149	167
**SAGc**	1	1	536	1	334	1	202	2	2	4	134	167	101

The total number of EtSAGs within each subfamily. The number of polymorphic genes, categorized by those exhibiting polymorphism in both the exon and intron (overall), the exon, or intron, along with the respective lengths (bp) of polymorphic exons and/or introns within these genes. The total number of variants detected within the exon and intron regions. The average polymorphism per base pair was calculated based on the length (bp) / number of variants detected in exon and/or intron.

Supplementary Table S6. Summary of EtSAG genomic sequences containing polymorphisms, their total length (bp), and average polymorphism per bp

	Number		Total length (bp)	Number of variant			Polymorphism per bp
	Total	Polymorphic		SNP	InDel	Total	
**Exon**	382	55	10,917	70	-	70	156
**Intron**	295	48	9,336	54	8	62	151
**Inter-coding region**	75	60	227,176	285	19	304	747
